# TECNOB: study design of a randomized controlled trial of a multidisciplinary telecare intervention for obese patients with type-2 diabetes

**DOI:** 10.1186/1471-2458-10-204

**Published:** 2010-04-23

**Authors:** Gianluca Castelnuovo, Gian Mauro Manzoni, Paola Cuzziol, Gian Luca Cesa, Cristina Tuzzi, Valentina Villa, Antonio Liuzzi, Maria Letizia Petroni, Enrico Molinari

**Affiliations:** 1 Istituto Auxologico Italiano IRCCS, Psychology Research Laboratory, Ospedale San Giuseppe, Verbania, Italy; 2 Department of Psychology, University of Bergamo, Bergamo, Italy; 3 Department of Psychology, Catholic University of Milan, Milan, Italy; 4 Istituto Auxologico Italiano IRCCS, Laboratory of Nutrition Research, Ospedale San Giuseppe, Verbania, Italy; 5 Istituto Auxologico Italiano IRCCS, Diabetes Research Laboratory, Ospedale San Giuseppe, Verbania, Italy

## Abstract

**Background:**

Obesity is one of the most important medical and public health problems of our time: it increases the risk of many health complications such as hypertension, coronary heart disease and type 2 diabetes, needs long-lasting treatment for effective results and involves high public and private costs. Therefore, it is imperative that enduring and low-cost clinical programs for obesity and related co-morbidities are developed and evaluated.

**Methods/Design:**

TECNOB (TEChnology for OBesity) is a comprehensive two-phase stepped down program enhanced by telemedicine for the long-term treatment of obese people with type 2 diabetes seeking intervention for weight loss. Its core features are the hospital-based intensive treatment (1-month), that consists of diet therapy, physical training and psychological counseling, and the continuity of care at home using new information and communication technologies (ICT) such as internet and mobile phones. The effectiveness of the TECNOB program compared with usual care (hospital-based treatment only) will be evaluated in a randomized controlled trial (RCT) with a 12-month follow-up. The primary outcome is weight in kilograms. Secondary outcome measures are energy expenditure measured using an electronic armband, glycated hemoglobin, binge eating, self-efficacy in eating and weight control, body satisfaction, healthy habit formation, disordered eating-related behaviors and cognitions, psychopathological symptoms and weight-related quality of life. Furthermore, the study will explore what behavioral and psychological variables are predictive of treatment success among those we have considered.

**Discussion:**

The TECNOB study aims to inform the evidence-based knowledge of how telemedicine may enhance the effectiveness of clinical interventions for weight loss and related type-2 diabetes, and which type of obese patients may benefit the most from such interventions. Broadly, the study aims also to have a effect on the theoretical model behind the traditional health care service, in favor of a change towards a new "health care everywhere" approach.

**Trial registration:**

Current Controlled Trials ISRCTN07265661

## Background

Since 1997, when the World Health Organization (WHO) described obesity as a chronic pathology with such an increasing incidence that it can be defined as a global epidemic, obesity is still an increasingly worrying issue for public health authorities. In 2005, about 1.6 billion adults (above 15 years of age) were estimated to be overweight, whereas about 400 million people were obese. WHO further projects that by 2015, approximately 2.3 billion adults will be overweight and more than 700 million will be obese [1]. Obesity is associated with early death [2,3] and is universally recognized as a risk factor for many health complications such as cardiovascular diseases, some types of cancer, osteoarthritis, hypertension, dyslipidemia, hypercholesterolemia and diabetes. Obesity is a strong risk factor for the development of type II diabetes [4,5]. Indeed, as BMI (Body Mass Index) increases, the risk of developing type 2 diabetes increases in a "dose-dependent" manner [6,7]. The prevalence of type 2 diabetes is 3-7 times higher in obese than in normal-weight adults, and those with a BMI >35 are 20 times more likely to develop type 2 diabetes than those with a BMI between 18.5 and 24.9 [8,9].

Given that obesity and associated type 2 diabetes lead to serious health consequences that in turn weigh heavily on public health care costs, developing effective interventions for substantially reduce weight, maintain weight loss and prevent or manage associated diseases like type 2 diabetes is compelling.

Stand-alone and combined treatment options (dietetic, nutritional, physical, behavioral, cognitive-behavioral, pharmacological, surgical) are available for both clinical and community populations, but clinical practice and research have shown significant difficulties with regard to availability, costs, treatment adherence and long-term efficacy [10]. From a public health strategy point of view, many problems are due to the high costs of these procedures, overall within an enduring care setting. Indeed, the main challenge in the treatment of obesity is to maintain weight loss in the long term [11]. Most overweight and obese individuals regain about one third of the weight lost with treatment within 1 year, sometimes even before the end of the intervention, and they are typically back to baseline in 3 to 5 years [12-14]. Similarly, few patients with diabetes go on taking their prescribed medication entirely as intended [15,16].

As suggested by Katan (2009) with regard to dieting, cognitions and feelings have a huge impact on behavior and may thus strength as well as disrupt adherence to treatment and compliance with clinical prescriptions. Indeed, psychological factors and processes mediate every behavior change and differently affect both the initiation and maintenance phases [17]. According to Ryan et al. [18], there are many approaches that have proved to be effective in initiating change, from external pressure and control to the positive use of incentives or rewards, but the ingredients essential to maintenance are still missing.

Enduring and cost-effective approaches that can reach broad populations of obese people are thus needed and have to be evaluated overall with regard to compliance and healthy behavior maintenance in the long term. A new promising method for granting continuity of care to wide populations of patients at low costs is telemedicine and its more specific branches called "e-therapy", "telecare" and "e-health": information and telecommunication technologies (ICT) used in order to exchange information useful for the diagnosis, treatment, rehabilitation and prevention of diseases [19,20]. Telecare can be carried out with tools such as web-sites, e-mail, chat lines (e.g. IRC, internet relay chat), videoconference, telephone and UMTS-based mobile-phone [21]. As already indicated in several studies [22-26], treatments delivered by ICT may be valid alternatives to reduce expensive and time-consuming clinical visits and to improve adherence to prescribed treatment through extensive monitoring and support.

For these reasons, we developed TECNOB (TEChnology for OBesity), a comprehensive two-phase stepped down program enhanced by telecare for the long-term treatment of obese people with type 2 diabetes seeking intervention for weight loss [27]. The core aspects of TECNOB are the hospital-based intensive treatment and the continuity of care at home using new information and communication technologies (ICT) such as internet and mobile phones. ICT-based approaches have already been shown to be helpful in weight loss and long-term maintenance [10,12,28-32], but much more work remains to be carried out in order to confirm these findings. Furthermore, very few studies have investigated the effects of an ICT-based program on obese patients with established type 2 diabetes and to our knowledge no study has tested a comprehensive long-term stepped down intervention starting with 1-month hospitalization specifically addressed to weight loss.

TECNOB protocol was mainly framed within the self-determination theoretical model of behavior change [33] and thus emphasizes the importance of maximizing the patients' experience of autonomy, competence and relatedness for the successful maintenance of behavior change [18].

This paper describes the design of the TECNOB study, a two-arm randomized controlled trial (RCT). The aims of this study are to evaluate the effectiveness of the TECNOB program in a sample of obese people with type 2 diabetes seeking treatment for weight reduction and to find out what behavioral and psychological variables are predictive of treatment success.

## Methods/Design

### Design

The effectiveness of the TECNOB program will be assessed in a two-arm randomized controlled trial. Participants will be randomly allocated in 2 groups:

1) TECNOB group: in-hospital treatment (diet, physical activity, psychological and dietitian counseling) plus extensive outpatient telecare through a web-platform and mobile phones;

2) CONTROL group: in-hospital treatment (diet, physical activity, psychological and dietitian counseling) and follow-up assessment at the 3rd, 6th and 12th month;

The Medical Ethics Committee of Istituto Auxologico Italiano approved the study protocol.

### Intervention

The TECNOB clinical program has a total duration of 13 months and consists of two stepped down phases: in-patient (1 month) and out-patient (the following 12 months). During the in-patient phase, participants undergo an intensive four-week hospital-based and medically-managed program for weight reduction and rehabilitation. Along this period, participants live in a medical hospital-like environment located on a mountain highland and far away from towns and cities. Visits from parents are allowed only in the afternoon. All patients are placed on a hypocaloric nutritionally balanced diet tailored to the individual after consultation with a dietitian (energy intake around 80% of the basal energy expenditure estimated according to the Harris-Benedict equation and a macronutrient composition of about 16% proteins, 25% fat and 59% carbohydrates). Furthermore, they receive nutritional counseling provided by a dietitian, psychological counseling provided by a clinical psychologist and have physical activity training provided by a physiotherapist.

Nutritional rehabilitation program aims to improve and promote change in eating habits and consists of both individual sessions (dietary assessment, evaluation of nutrient intake and adequacy, nutritional status, anthropometric, eating patterns, history of overweight, readiness to adopt change) and group sessions (45 minutes each twice a week) including: information on obesity and related health risks, setting of realistic goals for weight loss, healthy eating in general, general nutrition and core food groups, weight management and behavior change strategies for preventing relapse).

Psychological counseling is provided once a week both individually and in group setting. Individual sessions, lasting 45 minutes each, are mainly based on the cognitive-behavioral approach described by Cooper and Fairburn [34] and emphasize the techniques of self-monitoring, goal setting, time management, prompting and cueing, problem solving, cognitive restructuring, stress management and relapse prevention. Group sessions ("closed" groups of 5/6 persons), lasting 1 hour each, focus on issues such as motivation, assertiveness, self-esteem, self-efficacy and coping.

Physical activity takes place once a day except for week-end and consists of group programs (20 subjects) based on postural gymnastics, aerobic activity and walks in the open. Inpatients with specific orthopedic complications carry out individual activities planned by physiotherapists and articulated in programs of physical therapy, assisted passive and active mobilization and isokinetic exercise.

Low to moderate weight losses are expected at the end of the in-patient phase, but it is important to note that weight loss is not the primary goal of the in-patient program and each patient is made clear about this point at the very beginning of the treatment. Beyond the medical management of metabolic risk factors for health such as type 2 diabetes, developing a sense of autonomy and competence are the primary purposes of the in-hospital interventions. Patients are afforded the skills and tools for change and are supported in assigning positive values to healthy behaviors and also in aligning them with personal values and lifestyle patterns.

In the last week, just before discharge from hospital, participants are instructed for the out-patient phase of the program. They receive a multisensory armband (SenseWear^® ^Pro2 Armband), an electronic tool that enables automated monitoring of total energy expenditure (calories burned), active energy expenditure, physical activity duration and levels (METs) and sleep/wake states duration. Patients are instructed to wear this device on the back of the upper arm and to record data for 36 hours every two weeks in a free-living context. The Armband holds up to 12 days of continuous data which the outpatients are instructed to download into their personal computer and to transmit online to a web-site specifically designed for data storing. Outpatients are also told that they can review their progress using the InnerView^® ^Software which analyzes and organizes data into graphs and reports. Participants are then instructed to use the TECNOB platform, an interactive web-site developed by TELBIOS S.P.A. http://www.telbios.it. The TECNOB web-platform supports several functions and delivers many utilities, such as questionnaires, an animated food record diary, an agenda and a videoconference virtual room. In the "questionnaires" section, patients fill in the Outcome Questionnaire [35] and submit data concerning weight and glycated hemoglobin. In the "food record diary" participants submit actual food intake day by day through the selection of food images from a comprehensive visual database provided by METEDA S.P.A. http://www.meteda.it. The same procedure is also possible through a software called METADIETA (Meteda s.p.a.) previously installed on the outpatients' mobile phones before discharge. Through the mobile phones outpatients maintain the contact with the dietitian who regularly sends them SMS containing syntax codes that METADIETA, the software previously installed into the outpatients' mobile phones, uses in order to visually display the food choices (frequency and portions) outpatients have to adhere according to diet prescriptions. By this way, outpatients can keep a food record diary allowing comparisons between current eating and the recommended hypocaloric diet along the whole duration of the program. The "agenda" allows the patients to remember the videoconference appointments with the clinicians and the days when to fill in the questionnaires. Moreover, the patients can use the "memo" space to note down any important event occurred to him/her in the previous week/month. Indeed, some research indicates that changes in behavior (eating and exercise) often follow discrete moments which have been variably described as life events, life crises, teachable moments or epiphanies [36]. Life events can lead to weight loss but also to weight gain and qualitative research shows that it is not the event per se that results in behavior change but the ways in which this event is appraised and interpreted by the individual [37]. The clinical psychologist has thus the opportunity to discuss with the outpatients about the significant events reported in the "memo" space during the videoconference sessions and cognitively reconstruct dysfunctional appraisals in functional ways. Finally, outpatients are instructed to use the videoconference tool. Thanks to this medium, they receive nutritional and cognitive-behavioral tele-counseling with the dietitian and the clinical psychologist who attended the patients inside the hospital. In particular, just after discharge, participants have 6 videoconference contacts with both clinicians along 3 months. From the 3rd to the 6th month sessions are scheduled every 30 days and then even more spaced up to an interval of 60 days. During tele-sessions, clinicians (psychologist and dietitian) test the outpatients' progress, their mood, the maintenance of the "good alimentary and physical activity habits", the loss/increase of weight and ask about critical moments, especially those ones reported on the "memo" web-space. In particular, tele-sessions with the clinical psychologist aim to consolidate strategies and abilities acquired during the in-patient phase, to improve self-esteem and self-efficacy, to support motivation, to prevent relapse and to provide problem-solving and crisis counseling. On the other hand, dietitian assesses adherence and compliance to dietary therapy with a special focus on normal eating behavior, sufficient fluid intake, hunger and fullness regulation, appropriate eating/etiquette (pace and timing of meals), slow rate of eating, and addresses critical points such as plateau in weight loss or lack of readiness to improve dietary habits.

In addition to videoconference, outpatients can further contact clinicians by e-mail. Indeed, each patient is given the possibility to join his clinician beyond the established videoconference contacts in case of urgency or emergency. According to the e-message's content, clinicians choose the most appropriate format for delivering feedback among e-mail or telephone. In order to avoid excessive dependence and to contain costs, a maximum number of 1 not scheduled contact a week is established a priori.

As described, in the outpatient phase of the TECNOB program great relevance is given to the clinicians-patient relationship as an important medium and vehicle of change [18]. After discharge, out-patients begin to experience the autonomy and competence to change they develop during the in-patient phase and inevitably face resistances and barriers. Thanks to videoconferences, out-patients are supported by the clinicians who attended them during the in-hospital phase in exploring resistances and barriers they experience and in finding functional pathways to cope. Furthermore, out-patients are helped to experience mastery in terms of the health behavior change that needs to be engaged.

Participants in the control group will receive the hospital-based treatment and will be asked to respond to the follow-up assessments. No contact will be maintained with them at home and no continuous care will be provided after discharge.

### Study population

#### Recruitment of the study population

All inpatients with obesity and type 2 diabetes in the age range of 18-65 years who are referred to the S. Giuseppe Hospital of the Istituto Auxologico Italiano for weight-loss treatment will be asked and screened for admission to the study.

#### Inclusion and exclusion criteria

Inpatients are eligible when they meet the following inclusion criteria: 1) age between 18 and 65 years; 2) obesity according to the WHO criteria (BMI ≥ 30); 3) type 2 diabetes mellitus; 4) Basic knowledge of informatics and 5) written and informed consent to participate. Exclusion criteria for the study are: 1) severe psychiatric disturbance diagnosed by DSM- fourth revised criteria (ref.); 2) concurrent medical condition not related to obesity.

SCID (Structured Clinical Interview for DSM-IV Disorders) I and II (First, Gibbon, Spitzer, Williams, & Smith Benjamin, 2007; First, Spitzer, Gibbon, & Williams, 2007) are used as screening tools for psychiatric disorders and are administered by an independent clinical psychologist as part of his work.

#### Randomization procedure

All participants will be randomly assigned to the intervention or control group. The randomization scheme will be generated by using the Web site Randomization.com http://www.randomization.com. Randomization will take place after the baseline measurements.

#### Sample size calculation

The sample size calculation is based on a difference in weight regain of 3 kg between the intervention and control group after 12 months. The standard deviation of weight regain for the control group is considered to be 6 [12]. Based on these data, 64 participants per group are needed to detect this difference with an alpha of 0.05 two-sided and a power of 0.80. We decided not to make assumptions on sidedness because the TECHNOB program may also have negative effects in comparison with the control group. A sample of 154 persons (n = 77 per group) is required taking into account a dropout rate of 20%.

#### Measurements

Primary outcome measure of the randomized trial is weight in kilograms. Secondary outcome measures are energy expenditure, glycated hemoglobin, binge eating, self-efficacy in eating and weight control, body satisfaction, healthy habit formation, disordered eating-related behaviors and cognitions, psychopathological symptoms and weight-related quality of life. Data will be collected at baseline, at discharge from the hospital (c.a. 1 month after) and after 3, 6 and 12 months from the end of the in-hospital treatment.

##### Height and weight

Height will be measured with a stadiometer and weight will be assessed with the participant in lightweight clothing with shoes removed on a balance beam scale. Data collected at follow-up time points will be self-reported. Weight and height will be used to calculate BMI (weight in kilograms divided by the square of height in meters).

##### Energy expenditure

Energy expenditure is estimated using a multisensory armband (SenseWear^® ^Pro_2 _Armband, BodyMedia, Inc.). A proprietary heat-flux sensor measures the amount of heat being dissipated by the body by measuring the heat loss along a thermally conductive path between the skin and a vent on the side of the armband. Skin temperature and near-armband temperature are also measured by sensitive thermistors. The armband also measures galvanic skin response (GSR - the conductivity of the wearer's skin) which varies due to physical and emotional stimuli. A two-axis accelerometer tracks the movement of the upper arm and provides information about body position. The armband also contains a radio and a data port, allowing both wireless transmission and communication as well as wired downloading of data. SenseWear^® ^Pro_2 _Armband is a cost-efficient and simple solution that can be applied outside the laboratory in a free-living environment to track energy expenditure, physical activity durations and levels, and lifestyle information. For more detailed information on the armband and the algorithms it implements, visit the web-site http://www.armband.it.

##### Glycated hemoglobin

According to the Consensus Statement on the Worldwide Standardization of the Hemoglobin A1C Measurement [38], the hemoglobin A1C (A1C) assay has become the gold-standard measurement of chronic glycemia for over two decades. Anchored in the knowledge that elevated A1C values increase the likelihood of the micro-vascular complications of diabetes (and perhaps macro-vascular complications as well), the assay has become the cornerstone for the assessment of diabetes care. In this study, we adopt the measurement method (concentration of only one molecular species of glycated A1C) and results reporting (mmol/mol and derived NGSP %) developed by the International Federation of Clinical Chemistry and Laboratory Medicine (IFCC) [39].

### Psychological and behavioral questionnaires

Participants will complete the following questionnaires at entry to the study, at discharge from the hospital and at 3, 6 and 12-month follow-up time points by postal mail, Differently, the Outcome Questionnaire will be administered electronically through the web-platform before each videoconference session with the clinical psychologist.

#### The Self-Report Habit Index (SRHI) - Italian translation

The SRHI is a measure of the development and strength of habits. It has a stem " [the behavior] is something that ..." followed by 12 items such as "I do without thinking'. The SHRI has high internal consistency (α > 0.9), high test-retest reliability (r = 0.91) and high convergent and discriminative validity [40]. In our study, behaviors are: "eating in accordance with the prescribed diet" and "undertaking regular physical activity".

#### Weight Efficacy Life Style Questionnaire (WELSQ) - Italian version

The WELSQ is composed of 20 items that measure the confidence of the subjects about being able to successfully resist the desire to eat. The questionnaire was used to predict both weight loss and weight loss maintenance across a range of ages in men and women [21].

#### Body Uneasiness Test (BUT) - Italian version

The BUT is a self-report inventory that measures body uneasiness by a global severity index and five sub-scales: Weight Phobia, Body Image Concerns, Avoidance, Compulsive Self-Monitoring, Depersonalization [41].

#### Binge Eating Scale (BES) - Italian version

The BES is a short self-report questionnaire which measures severity of binge eating [42]. The Italian version of the instrument [43] consists of 16 items, which are composed by three or four sentences about the severity of binge eating. Cut-off score for mild binge eating symptoms is 17; scores between 18-26 indicate moderate binge eating symptoms and scores over 27 can be associated with a severe binge eating disturbance.

#### Eating Disorder Inventory (EDI-2) - Italian version

The EDI-2 is a widely used, standardized, self-report measure of psychological symptoms commonly associated with anorexia nervosa, bulimia nervosa and other eating disorders. The EDI-2 does not yield a specific diagnosis of eating disorder. It is aimed at the measurement of psychological traits or symptom clusters presumed to have relevance to understanding and treatment of eating disorders. The EDI-2 consists of 11 subscales derived from 91 items. Three of the subscales were designed to assess attitudes and behaviors concerning eating, weight and shape (Drive for Thinness, Bulimia, Body Dissatisfaction) and the remaining eight ones tapped more general constructs or psychological traits clinically relevant to eating disorders (Ineffectiveness, Perfection, Interpersonal Distrust, Interoceptive Awareness, Maturity Fears, Asceticism, Impulse Regulation and Social Insecurity) [44,45].

#### Symptom Check List (SCL-90) - Italian version

The SCL-90 is a brief, multidimensional self-report inventory designed to screen for a broad range of psychological problems and psychopathological symptoms. It consists of 9 symptom scales (Somatization, Obsessive-Compulsive, Interpersonal Sensitivity, Depression, Anxiety, Hostility, Phobic Anxiety, Paranoid Ideation and Psychoticism) and 3 global indices [46].

#### Impact of Weight on Quality of Life-Lite (IWQOL-Lite) - Italian version

IWQOL-Lite is the short version of the original IWQOL and is composed by 31 items. The questionnaire is self-report and consists of 5 scales assessing the impact of weight on QoL-related factors such as Physical Functioning, Self-Esteem, Sexual Life, Public Distress and Work. IWQOL-Lite has shown high internal consistency and high test-retest reliability [47,48]

#### The Outcome Questionnaire (OQ 45.2) - Italian translation

The OQ 45.2 is an self report questionnaire developed by Michael Lambert in 1996 [35]. The OQ 45 items version is a measure of outcome and it is designed in order to collect repeated measures of patient progress during therapy and after its conclusion. This instrument is one of the most used in psychotherapy research in the U.S. [49]. The OQ 45.2 is composed by 45 items that form 3 scales: Symptom Distress (SD), Interpersonal Relations (IR) e Social Role (SR), and a Global Index.

### Statistical analysis

Descriptive statistics (means ± SD, or median and interquartile ranges, as appropriate) will be used to describe the study sample with regard to baseline characteristics. Before selecting the most appropriate statistical tests, assumptions for parametric analyses will be checked. Repeated-measure ANCOVA will be used in order to evaluate the effects of the intervention when data do not violate the parametric assumptions. The mean differences between intervention and control group with 95% confidence intervals will be calculated. Analyses will be adjusted for possible confounders such as gender and age. Also effect modification will be investigated using interaction terms between intervention group and gender and age, respectively. If data violate parametric assumptions, we will use the exact methods with Monte Carlo approximation, a series of non-parametric statistical algorithms that enable researchers to make reliable inferences when data are sparse, heavily tied or unbalanced, not normally distributed, or fail to meet any of the underlying assumptions necessary for reliable results using the standard asymptotic method [50]. The Mann-Whitney test with Monte Carlo approximation will be used for independent measures, the Wilcoxon rank-sum test for repeated measures and the Fisher exact test for categorical variables. Weight data will be analyzed with an intention-to-treat (ITT) approach with dropouts assumed to have regained 0,3 kg per month, an assumption already used in previous studies [12,51]. Differently, missing data in the other variables will be replaced with baseline observation carried forward (BOCF) or last observation carried forward (LOCF) as appropriate, assuming no improvement for non-responders patients. Odds ratios with 95% confidence intervals will be also calculated at each follow-up time-point with respect to: 1) the percentage of participants maintaining or improving weight lost at discharge and 2) the percentage gaining a 5% and a 10% of baseline weight reduction for the TECNOB group in comparison with the control group. Finally, logistic regression will be used in order to find out predictors of treatment success defined as weight loss maintenance at the final follow-up time-point.

A confirmatory statistical test with alpha = 0,05 two-sided will be used for the primary outcome (weight loss in Kilograms), whereas explorative statistical tests will be used for all the secondary outcomes. Given the exploratory feature of the latter tests, critical alpha will be maintained at 0,05 two-sided without any correction for multiple comparisons.

All data analyses will be performed using the Statistical Package for the Social Sciences (version 12.0; SPSS, Inc., Chicago, IL).

## Discussion

The TECNOB program will be evaluated in a randomized controlled trial. We will test the effectiveness of this stepped down program enhanced by telecare on weight loss, weight loss maintenance, energy expenditure, glycated hemoglobin, binge eating, self-efficacy in eating and weight control, body satisfaction, healthy habit formation, disordered eating-related behaviors and cognitions, psychopathological symptoms and weight-related quality of life. Furthermore, we will explore the key behavioral and psychological variables predictive of treatment success and thus define the type of patients that benefit the most from such an intervention.

The theoretical approach behind the TECNOB program is to "move the healthcare where it really needs" using an advanced telemedicine web-platform and mobile phones to ensure the continuity of care at home. The core value of the program is overall the clinical use of mobile devices in the out-patients' everyday life. Indeed, the mobile connectivity can extend the treatment till the real-life environments of each patient, where traditional interventions typically fails because of the low compliance many obese patients have in carrying on with diet programs without active monitoring and support. Furthermore, mobile phones allow patients to receive "clinically oriented" SMS (such as food images indicating their day diet) that improve the reliability of the nutritional prescriptions. The results of this study will contribute to the evidence-based knowledge of how telemedicine may enhance the long-term efficacy of clinical interventions for weight loss and which type of obese patients may benefit the most from such an intervention. Results will also have an impact on the theoretical model behind the traditional health care service and, if positive, will promote a change towards a new "health care everywhere" approach.

## Competing interests

The technological devices described in this article, except for the web-platform, are commercially available and the authors declare not to hold on any financial interest therein.

## Authors' contributions

GC conceived the study, participated in its design and coordination, and helped to draft the manuscript. GMM participated in the study design and made substantial contribution to the manuscript drafting. PC, GLC, CT, VV participated in the study design, helped to draft the manuscript and revised it critically. AL, MLP, EM participated in the study design and coordination, and helped to draft the manuscript. All authors read and approved the final manuscript.

## Pre-publication history

The pre-publication history for this paper can be accessed here:

http://www.biomedcentral.com/1471-2458/10/204/prepub
